# Dr. Thomson on the Formation of Clouds

**Published:** 1830-10-01

**Authors:** 


### XXVI.

### ON THE FORMATION OF CLOUDS. By DR. THOMSON

IN a very able volume on Heat and Electricity, lately published by Prof. Thomson of Glasgow, we have some ingenious observations on the formation of clouds, vapour, fogs, &c. He observes that though the presence of vapours cannot be detected in the atmosphere by their colour, yet we can easily judge when the air is loaded with them, because we can see to a much greater distance, and the outline of the distant mountains is much more distinct than when the air is dry.

"In this country it is a comparatively rare thing to see the sky perfectly transparent. Much more frequently the vapour assumes a visible form, or becomes that opaque fleecy looking matter which we denominate a *cloud*. Sometimes these clouds are very high, very thin, and very small in size. At other times they sink lower down and envelope the whole face of the sky. Sometimes two or even three layers of clouds can be seen at different heights above each other. Whenever this happens, one of the layers is moving with more velocity than the others, or we observe one layer moving in one direction, and another in a different one. Sometimes these clouds sink down to the very surface of the earth and envelope it, in which case they are known by the name of *mists* or *fogs*.

There seems little reason to doubt that the cause to which Dr. Hutton ascribed the formation of rain, is in reality the cause of the formation of clouds. Dr. Hutton showed, by experiment, that when air of different temperatues, each containing as much vapour as is compatible with the temperature, are mixed together, a precipitation of moisture always takes place."

Now whenever moisture is precipitated from air, in consequence of the mixture of air of different temperatures, this precipitated moisture always assumes the appearance of a *cloud* or *mist*. We have a good example in the cloud formed when steam issues from the spout of a boiling tea-kettle. The steam has the temperature of 212°, and —we shall suppose the air at 60°, and saturated with moisture.

Force of vapour at 60° is 0·52 inch
 Do. do. 212° is 30
The mean of which is 15·26

But the force of vapour at 136°, the intermediate temperature, is only 5·14 inches. Hence a great proportion of the vapour, must be precipitated. But this precipitated vapour, instead of assuming the form of drops of water, which one would have expected, is converted into a *cloud* or *mist*.

Now *mist* or a *cloud* consists not of solid drops; but of a multitude of very thin vesicles of water, enclosing some aerial substance within them, similar to the vesicles usually blown from soap suds. That this is the structure of clouds and mists was affirmed by Derham and others, his contemporaries. Derham informs us, that he examined them by means of a microscope, and found them to be vesicles. Indeed the vesicular structure is obvious from the circumstance of clouds continuing elevated at a considerable height in the atmosphere, and that fogs may be often seen elevating themselves up the sides of mountains. If clouds or fogs consisted of round *drops*, their precipitation would be rapid. For a drop whose diameter amounted to $\frac{1}{10000}$th of an inch would acquire a velocity of nine or ten feet per second. Whereas we see clouds hover at a small elevation for hours, and they can in consequence be transported from the sea, lake, river, or marsh, from which they are raised, far into the country or to the tops of mountains, where the requisite supply of moisture cannot be had any other way.

M. de Saussure, senior, while travelling in the Alps happened to be enveloped in a mist which was almost stagnant. He was astonished at the size of the drops, as he thought them, and at seeing them floating slowly past him without falling to the ground. Some of them were larger than the largest peas. Catching them in his hand he found them to be bladders, inconceivably thin, and he found upon examination that they all had this structure. Indeed the optical phenomena exhibited by clouds and mists, show clearly that they are of a vesicular structure. If clouds were composed of drops they would exhibit a rainbow every time the sun shone upon them, supposing the spectator placed between the sun and the cloud. No such appearance, however, ever takes place. But every person must have observed the *halos* which are formed when a portion of fog is interposed between our eye and the sun or moon. The same halo may be perceived when the cloud formed by the mixture of steam and air is interposed between our eye and a candle or the sun. According to the measurements of De Saussure, the diameter of the smallest of the vesicles, of which clouds are usually composed, is about $\frac{1}{4222}$, and of the greatest about $\frac{1}{2620}$ of an English inch."

We have no conception, however, in what way these vesicles are formed—nor is it easy to conceive why these vesicles are sometimes lighter, sometimes heavier than air—sometimes exactly of the same gravity.

"Clouds then are formed whenever two strata of air of different temperatures, and each saturated with moisture, are mixed together. The sky in this country is usually much freer from clouds when the wind blows from the east than when it blows from the west. Because the temperature of the east wind being low, there is less chance during its continuance of strata of different temperatures mixing together. The comparatively high temperature of the west wind renders the chance of an intermixture of air of different temperatures during its continuance much greater. Should a west wind blow in the upper regions of the atmosphere, while an east wind blows near the earth, the whole sky would become clouded, because the contiguous portions of the two strata mixing together would occasion a deposition of moisture."

We recommend this highly interesting volume to our readers.

---

